# Childhood food allergies: An evolutionary mismatch hypothesis

**DOI:** 10.1093/emph/eox014

**Published:** 2017-10-04

**Authors:** Paul W Turke

**Affiliations:** Turke & Thomashow Pediatrics, 7444 Dexter-Ann Arbor Road, Dexter, MI 48130, USA

**Keywords:** childhood food allergies, evolutionary mismatch, immune system tolerance, Paleolithic diets

## Abstract

For hominins living in the Paleolithic era, early food antigen exposures—*in utero* and throughout infancy—closely matched later exposures, and therefore immune system tolerance mechanisms evolved under the expectation of this condition being met. This predicts that the degree of mismatch between early and downstream food antigen exposures is a key variable underlying the development of childhood food allergies. Three historical periods are identified in which the degree of mismatch climbs from near zero to substantial, as we transition from one period to another. The first encompasses our long history as foragers; the second begins with the advent of farming and the third spans only the most recent two or three decades, and manifests from social changes driven largely by an explosion in access to information. Testable predictions are generated and evaluated in light of available evidence, and an approach for primary prevention of childhood food allergies is proposed.

## INTRODUCTION

Childhood food allergies are a rapidly growing public health problem. Their prevalence in the USA increased by 50% between 1997 and 2011, and peanut allergies, which can be particularly severe and persistent, more than tripled between 1997 and 2017 [1, 2]. Other developed countries have experienced similar upward trends, and as less developed countries expand their economies they also are beginning to see increases [3, 4]. Overall, 220–250 million people worldwide currently have allergies to one or more foods, and children comprise the majority of cases [5].

## APPROACHING AN EXPLANATION

Although it is common to think of allergic reactions as immune system mistakes, some almost certainly are not. Many of the exogenous compounds that we come into intimate contact with—including some that have been labeled allergens—are irritants, or otherwise potentially damaging, and they elicit a measured response that is probably adaptive [6, 7]. It is a fine line, however, and some allergic reactions clearly are overdone. Fatal anaphylaxis is the quintessential example, but even more limited responses are also potentially maladaptive, particularly when they are to foods, because even relatively mild symptoms (e.g. nausea) can lead to future avoidance of the ingested item. This would have been consequential to our Paleolithic ancestors, especially during periods in which calories were in short supply, and/or when the foods causing reactions comprised a broad nutrient or calorie dense group (e.g. tree nuts).

The upshot is this: the immune system’s ability to be properly tolerant of foods has been subjected to natural selection for millions of years, which predicts a well-honed functionality that should not be prone to frequent overreaction. So, what is the source of the recent surge in childhood food allergies? Why is such an important, intricately designed system suddenly overreacting so often, and so vigorously, to specific foods?

Here, I combine two disparate bodies of knowledge, immunological and anthropological, to identify a mismatch in dietary antigen exposures that has increased markedly over the same 20 or so years in which food allergies have soared. In turn, I propose that the degree to which this mismatch is present is a major determinant of whether a childhood food allergy will or will not manifest.

## CULPABLE FOODS

Before proceeding to the hypothesis and the evidence that bears on, it will be useful to have in mind the foods that cause most allergic reactions, and to discuss underlying variables that help determine which make the short list. In the USA, eight foods are responsible for ∼90% of childhood food allergies: peanuts, tree nuts, cow’s milk, chicken eggs, wheat, soy, fish and shellfish [2]. Other developed countries have similar lists. For example, over 90% of food allergies in Australia are due to nine foods—the eight of the USA, plus sesame [8].

To be a significant source of allergy, a food must be widely consumed, and it must possess molecular properties that are intrinsically allergenic, such as thermal stability and resistance to proteolysis [9]. A highly allergenic food that almost no one eats will not make the list, whereas as a food that is only moderately allergenic might if it is sampled by a sizable portion of the population.

## TOLERANCE, T CELLS, THE THYMUS AND A VULNERABILITY

The human immune system becomes armed and potentially dangerous beginning *in utero*, and by necessity tolerance mechanisms develop in unison to control the growing destructive potential of the young immune system [10–12]. Furthermore, there is growing evidence that the mechanisms that build and guide tolerance not only develop early but also function at their highest level early in the lifespan—during fetal development, and during infancy while being breastfed and introduced to first foods [13, 14].

But why has peak function been pushed to such young ages? One reason surely is that infants cannot survive for long without having produced regulatory and effector cells that are both properly tolerant and capable of limiting infection. But this is unlikely to be the entire explanation. The cortex of the thymus—which nurtures and develops critically important young T cells (see below)—is not just functional early, it is prodigiously functional very early. It is proportionally largest and its output of T cells is proportionally greatest in the fetus and neonate, and then both decline relatively precipitously with age [15]. I have suggested that a specific vulnerability in T cell development contributes to the evolution of these outcomes [16]. The following is a focused summary of that argument, along with some updated evidence.

T cells direct and coordinate many immune system functions, including the production of IgE, the antibody that mediates most food allergies. However, before becoming functional T cells migrate to the thymus, where they are presented with a wide variety of antigens by specialized antigen presenting cells. If they bind tightly in this context, their development is arrested and they die. This is called ‘negative selection’, and it is a sensible way to eliminate T cells prone to attacking self and other substances that should not be attacked, given one key condition: the thymus must be a relatively privileged place in which the antigen being presented is nearly always self, or otherwise benign.

It has become increasingly clear, however, that this important condition is not always met [17]. Microbes are known to infiltrate the thymus, and some are known to manipulate negative selection to their advantage by having their antigenic components presented within the thymus, which then deletes young T cells that upon maturation would have been capable of recognizing and destroying them. *Mycobacterium avian*, for example, is known to sometimes produce this devious outcome, and many other microbes, including *Mycobacterium tuberculosis* and the hepatitis B virus, are suspects [17].

Hosts have countered, I have suggested, by evolving the means to produce vast numbers of long-lived T cells when the thymus is least likely to be infiltrated by microbes. For humans, and most other mammals, this is while *in utero*, because multiple mechanical barriers, including the placenta, are very good at shielding fetuses from microbes, and the experienced maternal immune system also very effectively limits fetal exposure to pathogens. And since some of this maternally derived protection extends into the newborn period—maternally derived IgG persists for months, and secretory IgA and other immunity conferring substances are present in breast milk—I have argued that this also is a relatively good time of life in which to be building a repertoire of properly tolerant T cells [16].

Perfection, of course, is elusive in biology. Although the foregoing approach potentially mitigates the problem of microbial subversion of T cell development within the thymus, I will argue below that mistakes can occur when tolerances developed early in lifetimes don’t match well with those that are needed at older ages.

## PALEOLITHIC DIETS

If immune system tolerance develops in accordance with the above description, then it’s logical to suspect that the key to understanding the manifestation of food allergies is knowing how today’s food antigen exposures over individual lifetimes differ from those that evolving hominin immune systems counted on for million years. It is now possible to make such a comparison because our Paleolithic diets are increasingly well-known [18–21]. Below are three secure inferences, or rules of thumb, that are drawn from this growing body of data, and I suggest that a small number of specific sociocultural changes (identified below) have broken these rules, at first slowly and then rapidly, resulting in the surge in childhood food allergies cited in the Introduction.

Rule 1. Paleolithic diets usually were comprised of great variety. Direct evidence supports this inference, and it comports well with the widely held view that for millions of years our ancestors were opportunistic foragers [18–21].

Rule 2. Although our ancestors ate many different foods, it was almost always the same different foods year after year and lifetime and after lifetime. This inference also can be made with confidence because throughout all but the most recent portion of our evolution the introduction of novel foods hinged on events that occur slowly or infrequently, generally on evolutionary time scales. For instance, changes in climate, long-distance migrations to new eco-zones, the control of fire and the invention of new tools, all potentially generate dietary novelty but not rapidly or often [18–21].

Rule 3. Given Rule 2, throughout the Paleolithic era food antigen exposures encountered by fetal, infant and toddler immune systems would have closely matched subsequent exposures. Our tolerance mechanisms therefore evolved under the expectation that early exposures predict later exposures.

The mechanistic underpinnings of Rule 3 are 3-fold. First, antigens originating in maternal diets—including some that today are recognized as major allergens—are known to transfer readily to the fetus via the placenta [10, 22]. Second, it is well-established that peanut proteins can be secreted into breast milk, if the maternal diet includes peanuts, which suggests that exposure to at least some maternal dietary antigens can persist until weaning occurs, which for extant foragers—and by extrapolation, our distant ancestors—is at ∼2–3 years of age [10, 23]. And a third route of exposure of course is the direct consumption of solid foods by infants and toddlers, which also by extrapolation probably was initiated in ancestral populations at around 6 months of age [18].

Thus, by these three mechanisms, children alive in the Paleolithic era who reached the age of 1 year or perhaps a little older (i.e. 9 months *in utero* + 12–15 months postpartum) would have been exposed to most or perhaps even all of the different types of food antigens they would ever be exposed to. This stands in sharp contrast to the children of today who live in developed countries. They are at risk of an unprecedented degree of mismatch between early and late food antigen exposures, and I suggest that this is a significant cause of the recent, rapid increase in their food allergies. So, what has broken the rules, particularly Rule 3?

## FARMING, SOCIAL STRATIFICATION, TRADE, MOBILITY AND THE INTERNET

Farming is the initial precipitator. It began to bend and then break Rule 1 in the fertile crescents of the Middle East ∼12 000 years ago by trading the nutritional variety experienced by foragers for a narrower but more reliable source of calories, mostly from grains. This led to population growth and also, for many, diminished health [18–21, 24].

Farming also led to vast changes in social organization and behavior [25]. Populations became sedentary and more dense; social stratification and the creation of wealth disparities occurred on a scale unknown in foraging societies and long-distance trading of food (among other items) became a reality. It of course took thousands of years for farming and all that it portends to spread throughout the world, and as it did it initially introduced the possibility of eating novel foods primarily to a relatively small number of individuals at the top of the social hierarchy. Lower ranking individuals comprising the majority of the population saw their diets change by becoming narrower but they nonetheless remained provincial in most other respects. They did not travel widely, they did not share in the wealth that became available to the upper strata and therefore their access to new foods and other items through trade would have been minimal. However, the trend was established, and the Rules, above, never again would be followed to quite the extent that they were among our more distant ancestors.

**Figure 1.**
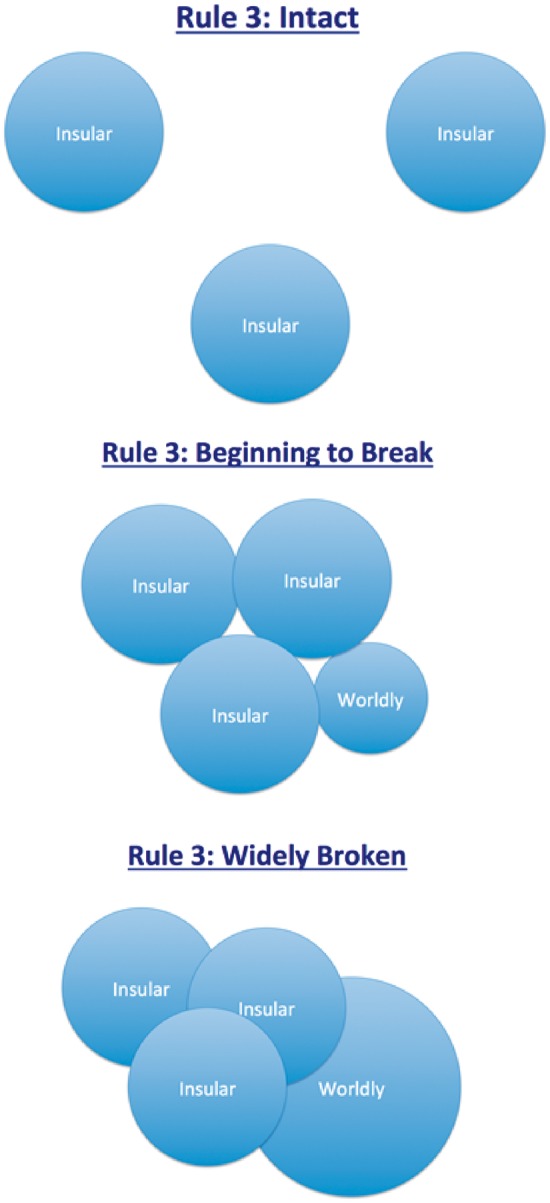
Dietary insularity and adherence to Rule 3 among foragers, farmers and Internet users

Figure 1 gives a pictorial view of this progression. It divides our existence as hominins into three periods that over time exhibit decreasing adherence to Rule 3 as a function of increasing interaction between formerly insular groups.

The first period shown at the top covers the long expanse of time during which we were foragers (aka Hunter-Gatherers). Circles demarcate regions in which bands of individuals interacted and shared food. The area of a given circle is not known with precision for any ancestral population, however, current and recent foragers (without access to horses) have ranges that encompass generally no more than a few hundred square miles [26, 27], which likely would have been near the maximum for our ancestors. The key point is that there would not have been interaction with foraging bands living at a distance in potentially different eco-zones, with different technologies and traditions and different diets, and therefore Rule 3 would have been intact.

The second period shown in the middle includes most of the post-farming era beginning 12 000 years ago and ending just prior to the start of the 21st century. Circles that were once separated are shown bumping into one another and beginning to overlap due to technological innovations, like the invention of the wheel and domestication of horses and camels, and also due to socioeconomic innovations like money that motivated and nurtured long-distance trade. Boats, trains, automobiles and planes eventually advanced this trend considerably, and wealth disparities, which barely existed among foragers, appeared and became extreme in some instances [28]. As a result, there arose for the first time a segment of the population that could aptly be called ‘worldly’. It was comprised of a small but growing number of individuals with the motivation, wealth and mobility to access foods grown and processed at a distance. For them, Rule 3 would be broken with increasing regularity.

The third period shown at the bottom extends to the present. It exploded into existence only ∼20–25 years ago and coincides with rapid growth of the segment of the population first referred to in the previous period as worldly. Its growth has been fueled not so much by access to wealth as by access to information. Personal computers and the Internet were the key innovations. They created the presciently named world-wide-web, which made a world that was already getting smaller much smaller almost overnight by making it easy for anyone in the developed world with the will to do so to peer into the lives of others—and this turned others into virtual neighbors.

Today, it’s easy to have recipes from far-away places streamed to a mobile device, say from one of the various 24-h food channels, and their ‘exotic’ ingredients also can be easily accessed. This ability to share dietary traditions is unprecedented, and accelerating. It, along with in-the-flesh ethnic and racial mixing, which also has accelerated in part because learning about one another motivates coming together, has dramatically increased the quality of life in so many ways for so many of us but it has also led to unprecedented breaches in Rule 3.

The problem, with respect to childhood food allergies, is that the mixing of dietary traditions is at a crossroads. Large numbers of people who’ve borrowed widely from the culinary traditions of others live among large numbers of individuals who have remained insular with respect to diet. For every foodie, world traveler, ethnic restaurant voyeur and adventurous grocery shopper, there is a friend, neighbor, relative or coworker who eats far more narrowly. Thus, a child who was not exposed to, for example, peanut or fish proteins during gestation, breastfeeding and in the natal home as an infant and toddler has a far greater chance than even two or three decades ago of being exposed to them downstream, when beginning preschool, kindergarten or just upon being invited to a neighbor’s house for lunch.

Although the trends outlined immediately above are to some extent self-evident, there are also a number of empirical studies that bear on them. Some are industry reports, and have a narrow focus that limits their value, but two comprehensive studies are particularly relevant, and will be briefly discussed [29, 30]. The first tracked dietary changes in the USA between 1999 and 2012 and found significant increases in the consumption of whole grains, nuts, seeds, fish and shellfish. It is notable that the reported increase in consumption of these five foods—which happen to be among those responsible for many allergic reactions—did not occur uniformly. Some individuals and groups resisted change, whereas others embraced it, which should increase the risk of the type of mismatch described above. The second study found that ethnic food consumption in the USA has been accelerating rapidly for more than a decade, fueled, the authors state, both by increasing ethnic diversity in the USA and increasing international travel. These trends are important to note because imported cuisines often include novel ingredients, thus potentiating dietary mismatch, and because many, particularly those with Asian roots, tend to include foods known to trigger allergic reactions in children.

Thus, to briefly summarize, until very recently Rule 3 has been sufficiently intact to keep the prevalence of food allergies largely in check. But no longer, and therein lies my proposed explanation for the recent surge in childhood food allergies.

## PREDICTIONS AND CURRENT EVIDENCE

The central prediction of the ‘mismatch hypothesis’ is that childhood food allergies will increase as violations of Rule 3 increase. There are many potential avenues for testing this prediction but they are not equally feasible or definitive. For example, it is straightforward to predict an increase in childhood food allergies at the boundary of the Pleistocene-Holocene transition in regions where farming and long-distance trade were first adopted. However, since there are no prehistorical accounts of food allergies, and only anecdotal accounts in the distant historical record, this specific prediction cannot be tested directly. It might be possible, however, to get a glimpse of what happened in the past by looking at extant foragers and determining whether childhood food allergies increase when formerly isolated groups come into contact with the world at large, and thereby are exposed to novel foods. Some such groups have been extensively studied (e.g. Kung, Ache and Hadza) but I am unaware of any published relevant data.

Furthermore, children emigrating from one country to another at a very young age, and children born soon after their mothers emigrated, are predicted to have more food allergies than their age-matched compatriots, under the assumption that in these circumstances they will be exposed primarily to food antigens from their homeland until they reach an age where they interact outside the natal home, and in turn risk dietary mismatch. Support can be construed from a large comparative study that found foreign-born children arriving in the USA before age two are at significantly greater risk of developing a food allergy than children arriving at older ages, and children born to very recent immigrants are at even higher risk [31].

Although broad population-level comparisons like the above can be informative, there are several recent studies that more directly, and more rigorously, test the mismatch hypothesis. They address both pre- and postpartum introduction of allergenic foods in age-matched cohorts.

One of these, a prospective cohort study by Frazier et al. [32] looked at maternal consumption of peanuts and tree nuts during pregnancy and found a large and highly significant reduction in the development of allergies to peanuts and tree nuts in children whose mothers consumed them regularly, compared with those whose mothers avoided them while pregnant. Another study followed children introduced to peanuts beginning at 6 months of age [33]. It, too, found a large, highly significant protective effect, and a follow-up study by the same authors demonstrates that once tolerance to peanuts is established by early introduction, it persists even when they are avoided for over a year and then reintroduced [34]. And in yet another study, the early introduction of chicken eggs was shown to be protective compared with delayed introduction [35].

Notwithstanding the foregoing support, a recent article by Perkin et al. [36] goes against the proposition that early introduction is protective. They found in an experimental trial that exclusively breastfed babies randomized to consume cow’s milk, egg, peanut, sesame, white fish and wheat between three and 6 months of age were no less likely to become allergic to these foods than matched controls. It is important to emphasize, though, as this study’s authors have, that compliance within the early introduction group was only 39.1%, apparently because these very young babies resisted eating their assigned foods. When analysis was restricted to only individuals with good compliance, the study found a statistically significant protective effect from early introduction.

Overall, support for a protective effect from early introduction of allergenic foods is growing but contrary examples are not hard to find. For instance, Frazier et al. identify four retrospective studies that, opposite of their findings, suggest that peanut consumption in pregnancy increases the risk of children developing peanut allergy [30]. In the end, they discount these older studies on grounds of recall bias but is this justifiable? Allergists and pediatricians are not of one mind but changes in formal guidelines presumably reflect how conflicting empirical results have been reconciled over the years by leading experts.

## THE EVOLUTION OF FOOD ALLERGY PREVENTION GUIDELINES

In 2000, the American Academy of Pediatrics and similar organizations in Europe recommended strict avoidance of peanuts while pregnant; avoidance of peanuts, tree nuts and fish while breastfeeding and for infants themselves avoidance of all major allergenic foods prior to reaching 1 year of age, with two caveats: no eggs until age two, and no peanuts, tree nuts or fish until 3 years old [2].

In 2008, the recommendation to avoid allergenic foods during pregnancy and breastfeeding was rescinded. However, a complementary recommendation to eat allergenic foods during these critical periods was not made, and this null position is still in place. On the other hand, in a major reversal, direct consumption of allergenic foods by infants beginning at 4–6 months was added as a formal recommendation in 2008, and current guidelines continue to give this advice with some caveats based on comorbidities such as eczema [2].

Guidelines on peanut consumption are especially illustrative of the complexity of the problem. As of 2016–17, the National Institute of Allergy and Infectious Diseases recommends introduction of peanut containing foods any time after the introduction of other foods, if there is no eczema and if there has not been any allergic reaction to the other foods. If there is mild to moderate eczema, the recommendation is to introduce peanuts specifically at 6 months of age, after successfully introducing other foods. Finally, for infants with severe eczema, egg allergy, or both, the most up-to-date recommendation is to ‘consider’ first doing a skin prick test or measure serum IgE levels to peanut antigen. If there is a minimal skin pick reaction, or a low IgE level, peanuts should be introduced at home at 4–6 months of age or they should be introduced at a physician’s office at 4–6 months. No basis for choosing one approach over the other is given, but it presumably turns on the subjective judgment of the parents, or the physician who ordered the tests. For children with high IgE or skin prick responses, strict avoidance is recommended until such a time as further testing indicates that test results have moved into the lower range [2, 37].

It is likely that no one is completely satisfied with the current guidelines. They are complicated, subjective and silent on the question of consumption while pregnant and breastfeeding. More data are needed, and I suggest that a study along the following lines would go a long way toward filling in the blanks.

## A SUGGESTED STUDY

A group of newly expectant mothers—the interventional arm—would be exposed daily or at least several times per week throughout pregnancy and while breastfeeding to the short list of foods that cause most of the childhood food allergies in their country or region. This could be accomplished by regularly eating the most culpable foods, or, for the sake of conformity and compliance, by ingesting a capsule, or packet mixed with water, that contains their antigenic components. In addition, this same group of mothers would introduce these foods and/or a suitable antigen containing supplement directly to their infants beginning at age 4–6 months, and this would be continued, along with breastfeeding, for at least 1 year. The other side of the experiment would be to establish a control arm that is similar to the interventional arm, except that advice on what to eat, and when, would be derived from currently accepted guidelines. The children comprising the two arms would then be followed throughout childhood, or a significant portion of it, for the development of food allergies.

Finding no effect, or increased allergies in the interventional arm, would reject the mismatch hypothesis, whereas it would be strengthened if children in the interventional arm went on to develop fewer foods allergies than children in the control group. Ideally, supportive results would lead to further testing to determine optimal timing of exposures, as well as optimal dosing.

## DISCUSSION

The mismatch hypothesis does not purport to give a complete explanation of food allergies. It more modestly identifies a single variable, the degree of adherence to Rule 3, and uses it to explain primarily one event: the soaring prevalence of childhood food allergies in developed countries over the past 20 or so years.

The most salient alternative to the mismatch hypothesis is the hygiene hypothesis [38], which is now often referred to as the ‘old friends’ hypothesis [39]. The gist is that humans have a long-standing evolved relationship with the worms, bacteria, fungi and viruses that live on us and in us, and there is compelling evidence indicating that these old friends influence the immune system’s development and contribute to its regulation over the entire lifetime [40]. Thus, it’s been sensibly proposed that disruption of these ancient relationships by various factors—the use and abuse of antibiotics, increased consumption of refined sugars and new types of fats and migration from farms to cities—can potentiate allergic and autoimmune reactions. But, there is a timing problem. Widespread use of antibiotics has been ongoing for more than 50 years, our diets have been altered in ways that change the composition of our old friends for even longer, and farm to city migration has been underway for longer still. Thus, for the old friends hypothesis to be a stand-alone alternative to the mismatch hypothesis it would be necessary to make the case that a threshold was crossed only 20–25 years ago that so disrupted immune function that it finally caused the surge in childhood food allergies being addressed. That case has not yet been made. Furthermore, the old friends hypothesis cannot easily accommodate the findings reviewed above in which introducing allergenic foods early reduces the risk of developing a food allergy. For all of these reasons, I believe that the mismatch hypothesis delivers the key variable, albeit the disruptions proposed by the old friends hypothesis might nevertheless contribute to the rise of allergic and autoimmune diseases in a supporting role.

And it may be fortuitous that disruption of our ancient relationships with worms and microbes is not the most likely source of the recent surge in childhood food allergies. This is because antibiotics, processed foods and urban living are very entrenched, and therefore difficult to reverse, whereas the mismatch hypothesis maps out a simple remedy. It requires only that we shore-up Rule 3. This could be accomplished in principle by advising pregnant and nursing mothers, and their 4-to-6-month-old infants, to be regularly exposed to the antigenic components of the foods that cause most childhood allergies, either by eating the foods, taking a supplement, or both.

## CONCLUSION

In criminal investigations, evidence pointing to a particular suspect can be compelling in its own right but it becomes more compelling in the presence of a well thought out motive. My goal for the mismatch hypothesis is essentially the same. It helps us better understand the available evidence, it pinpoints areas where more is needed, and at this juncture it adds weight to the side of the argument in favor of early introduction of allergenic foods—but not just for infants who have reached the age of 4–6 months. It also weighs in favor of consuming allergenic foods during pregnancy and while breastfeeding. In contrast, the consensus view among allergists and pediatricians has been, since 2008, to abstain on this issue. Although the intention is to avoid giving bad advice, the hard reality is that millions of pregnant and nursing mothers either will, or will not, consume the foods that cause most childhood food allergies, and only one of these two courses of action is likely to be correct. It can be risky, in other words, to wait for the last shred of evidence to be gathered before taking action, and perhaps riskier still to make medical recommendations without first reflecting on the broadest of all theories of life: evolutionary theory.

**Conflict of interest**: The author has a patent pending on a process/product for the primary prevention of childhood food allergies. 
